# Induction of serine hydroxymethyltransferase 2 promotes tumorigenesis and metastasis in neuroblastoma

**DOI:** 10.18632/oncotarget.28168

**Published:** 2022-01-06

**Authors:** Rachael A. Clark, Jingbo Qiao, Jillian C. Jacobson, Dai H. Chung

**Affiliations:** ^1^Department of Surgery, University of Texas Southwestern Medical Center, Dallas, TX 75234, USA; ^2^Department of Surgery, Children’s Health, Dallas, TX 75234, USA

**Keywords:** neuroblastoma, serine hydroxymethyltransferase 2 (SHMT2), tumorigenesis, serine metabolism, one-carbon metabolism

## Abstract

High-risk neuroblastoma (NB) remains an extremely difficult subgroup to cure and is associated with *MYCN* amplification. Serine hydroxymethyltransferase 2 (SHMT2) regulates serine metabolism in a myc-dependent manner; it is upregulated in several cancers and is associated with tumor aggressiveness. Akt-2, an important regulator of *MYCN* via the PI3K/Akt pathway, induces metastatic potential in NB. The association between SHMT2 and PI3K/Akt in hepatocyte regeneration has been well established but its mechanistic interaction in cancer has yet to be clearly elucidated. Herein, we evaluated the exact role of SHMT2 on the PI3K/Akt pathway, in addition to NB tumorigenesis and metastatic potential *in vitro*. *SHMT2* gene expression and overall survival (OS) were assessed. Two human NB cell lines were examined. SHMT2 silencing and overexpression were performed. The downstream effects were analyzed with immunoblotting, RT-qPCR and functional assays were performed. We found *SHMT2* gene expression is associated with decreased OS and *MYCN* amplification. SHMT2 protein and mRNA expression are increased in *MYCN*-amplified cells. SHMT2 expression has a direct interaction with Akt-2 and *MYCN*. Induction of *SHMT2* increased cellular proliferation, colony formation and cellular migration and *SHMT2* expression was increased in metastatic NB cells. We conclude that SHMT2 regulates N-Myc via phosphorylation of Akt-2 and plays an important role in NB tumorigenesis by contributing to cell growth, migration, colony formation and metastasis *in vitro*.

## INTRODUCTION

Neuroblastoma (NB) is a pediatric tumor derived from neural crest cells. It is the most common pediatric, extracranial solid tumor. The high-risk group of NB remains one of the most difficult subgroups of NB to treat with resistance to therapeutic regimens and high disease relapse. Children with high-risk NB tumors have a long-term survival rate of less than 50%. High-risk disease is classified based on imaging stage, patient age, histology, presence of diploidy, 11q aberrations or *MYCN* amplification [1]. Surgical resection is a viable treatment option for patients with low-risk NB. However, high-risk NBs require multi-modality treatment including chemotherapy, radiation, stem-cell transplant and recently, immunotherapy. Despite these treatment advances, high-risk NB has a low disease-free survival and increased rate of relapse or remission with many tumors developing chemotherapy and radiation resistance [2].

Altered metabolism is a key feature of cancer cells and allows cells to survive in stressful conditions. Glucose and glutamine metabolism are commonly reprogrammed pathways with mutations involving the MYC family. *MYCN* encodes for the oncoprotein N-Myc. *MYCN* amplification and overexpression in NB is associated with increased proliferation and enhanced malignant potential, while knockdown of *MYCN* has been shown to result in tumor growth arrest and apoptosis in NB [3]. Increased MYC gene expression affects several aspects of metabolism such as glycolysis, mitochondrial function and serine metabolism [4].

Serine plays an important role in one-carbon metabolism as a one-carbon donor to the folate cycle [5]. Altered serine metabolism has been linked to several cancer types and has been shown to be affected in a myc-dependent manner by mitochondrial serine hydroxymethyltransferase 2 (SHMT2) [4]. SHMT2 converts serine to glycine in the mitochondria and is induced by HIF1α and MYC in hypoxia to promote cell survival. Upregulation of SHMT2 is found in many types of cancers, including lymphoma, glioma, cholangiocarcinoma and breast cancer [6, 7]. In both glioma and breast cancer, SHMT2 is associated with tumor aggressiveness and is an independent predictor of prognosis [8, 9].

Although little is known about the exact role of SHMT2 in NB, a recent report found an association between SHMT2, N-Myc expression and high-risk NB [10]. A commonly deregulated pathway involving N-Myc in high-risk NB is the phosphatidylinositol 3-kinase (PI3K)/Akt pathway, which regulates angiogenesis by stabilizing N-myc [11]. In addition, Akt-2 is an important regulator of NB metastatic potential. While the exact molecular interaction between SHMT2 and PI3K/Akt in malignancy has not yet been elucidated, another recent study demonstrated that increased expression of SHMT2 in hepatocytes led to physiologic Akt activation via PI3K [12]. Given the association between SHMT2 and PI3K/Akt in hepatocytes, and the importance of the PI3K/Akt pathway in NB angiogenesis and metastasis, SHMT2 may play a critical role in NB tumorigenesis and metastasis via the PI3K/Akt pathway. In the present study, we sought to determine the exact role of SHMT2 in the PI3K/Akt pathway and the effect of SHMT2 on NB tumorigenesis and metastasis *in vitro.*

## RESULTS

### SHMT2 expression is associated with *MYCN* amplification and decreased overall survival

**Figure 1:**
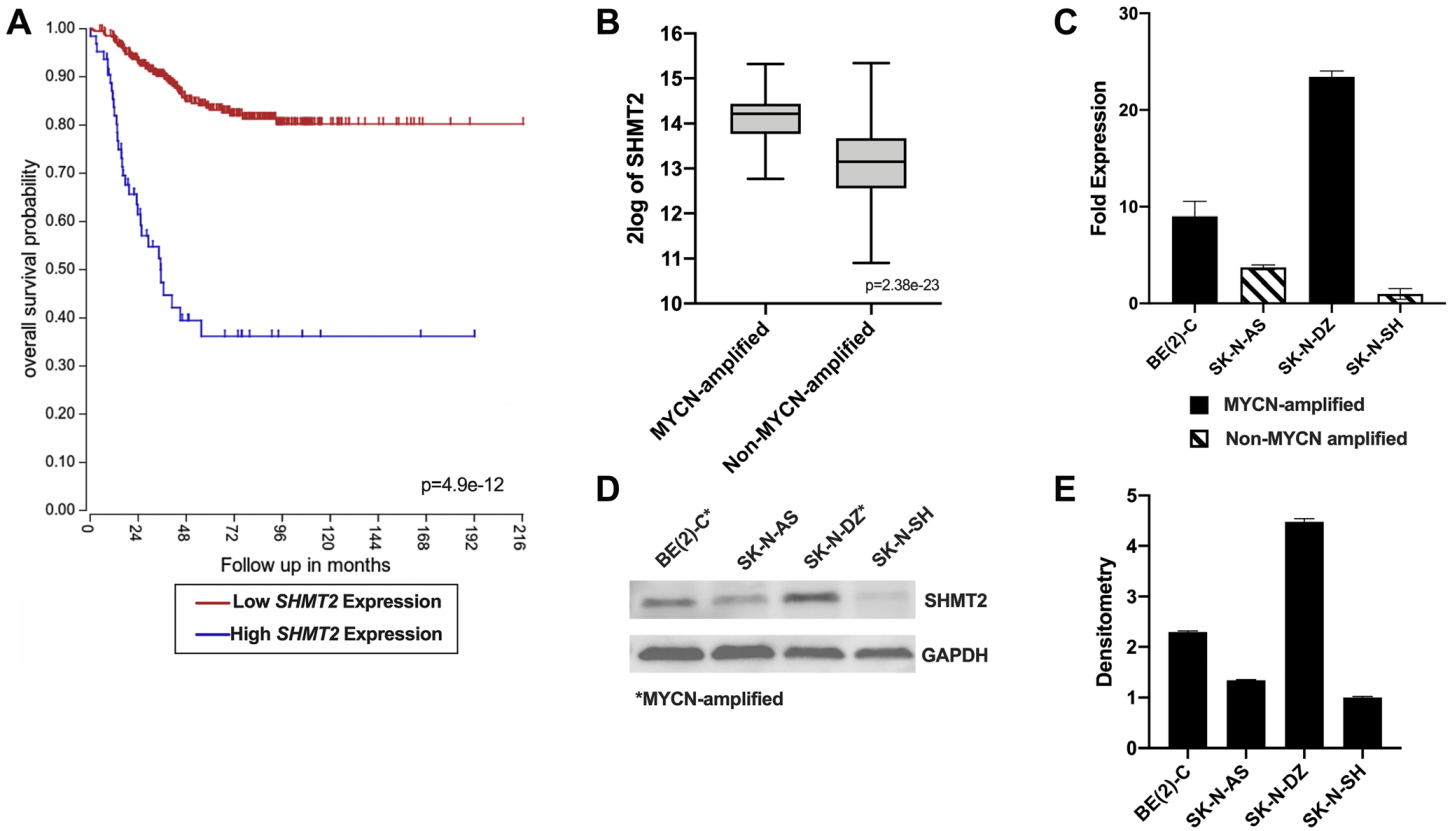
*SHMT2* gene expression is associated with decreased survival and *MYCN* amplification in neuroblastoma patients. (**A**) Kaplan-Meier survival curves created using the R2 genomics analysis application and database shows a decrease in overall survival with increased *SHMT2* expression in NB patients. (**B**) *SHMT2* gene expression is significantly higher in patients with *MYCN*-amplified NB compared to non-*MYCN*-amplified NB. (**C**) *SHMT2* mRNA expression is increased up to 23.4-fold in the *MYCN*-amplified cell lines, BE(2)-C and SK-N-DZ, compared to the non-*MYCN*-amplified cell line, SK-N-AS and SK-N-SH. (**D**) Immunoblotting demonstrated increased SHMT2 protein expression in the *MYCN*-amplified cell lines, BE(2)-C and SK-N-DZ, compared to the non-*MYCN*-amplified cell lines, SK-N-AS and SK-N-SH. (**E**) Densitometry analysis confirmed a 2.3 to 4.5-fold increase in SHMT2 protein in expression in the MYCN-amplified cell lines, BE(2)-C and SK-N-DZ, compared to non-*MYCN*-amplified cell lines, SK-N-AS and SK-N-SH.

Given the role of SHMT2 as an independent predictor of prognosis in breast cancer and glioma, we sought to determine the potential relationship between *SHMT2* gene expression and overall survival in NB patients [8, 9]. Using the R2 genomics analysis application to evaluate the Kocak database, consisting of 649 NB patients, the relationship between *SHMT2* expression and overall survival (OS) in NB was examined. The optimal cut-off value for high versus low expression is calculated in the R2 database using a KaplanScan, a logrank test that identifies the most significant expression value for survival analysis. The optimal gene expression cut-off value for high versus low *SHMT2* expression in the Kocak database was 19713.8 (Supplementary Figure 1). As shown in Figure 1A, increased *SHMT2* expression was associated with a significant decrease in OS in NB patients, with an OS of 39% in patients with high *SHMT*2 expression compared to an OS of 80% in patients with low *SHMT2* expression. In addition, the relationship between *MYCN* amplification and *SHMT2* expression in NB patients was also analyzed. We found an increased *SHMT2* expression in patients with *MYCN* amplification compared to those without *MYCN* amplification (Figure 1B).

SHMT2 mRNA and protein expression were evaluated in four NB cell lines: two *MYCN*-amplified cell lines, BE(2)-C and SK-N-DZ, and two non-*MYCN*-amplified cell lines, SK-N-AS and SK-N-SH. SHMT2 mRNA expression was assessed using reverse transcription-quantitative polymerase chain reaction (RT-qPCR). Both *MYCN*-amplified cell lines, BE(2)-C and SK-N-DZ, demonstrated a 2.4 to 23.4-fold increase in *SHMT2* mRNA expression compared to the non-*MYCN*-amplified cell lines, SK-N-AS and SK-N-SH (Figure 1C). SHMT2 protein expression was evaluated using immunoblotting and quantified with densitometry analysis. SHMT2 protein expression was increased in both of the *MYCN*-amplified cell lines, BE(2)-C and SK-N-DZ, and decreased in the non-*MYCN*-amplified cell lines, SK-N-AS and SK-N-SH (Figure 1D). Densitometry analysis demonstrated a 1.8 to 4.5-fold increase in SHMT2 protein expression in *MYCN*-amplified cells compared to the non-*MYCN*-amplified cells (Figure 1E).

### SHMT2 regulates N-Myc via decreased activation of Akt-2

**Figure 2:**
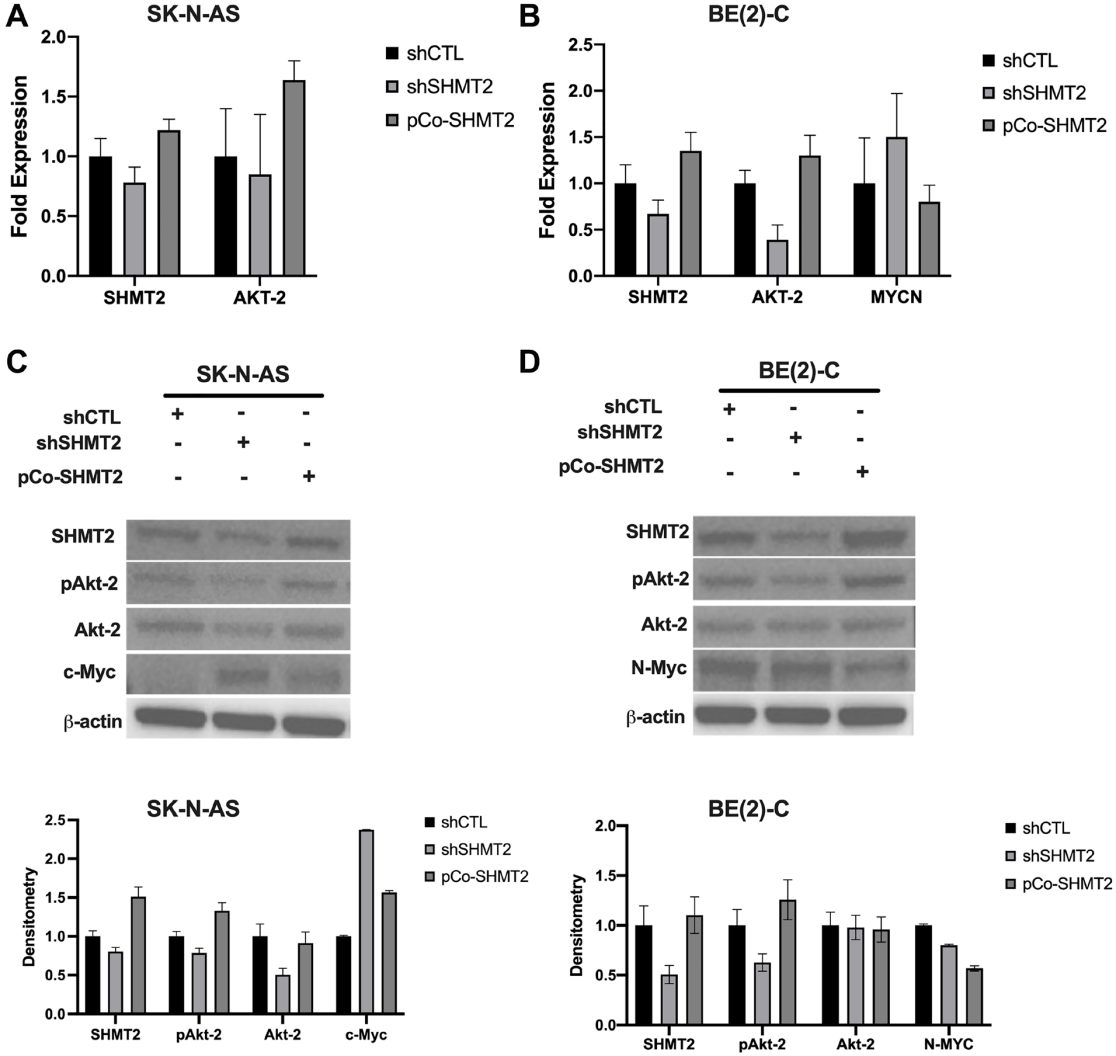
SHMT2 regulates N-MYC via decreased activation of Akt-2. RT-qPCR and immunoblotting were used to assess pAKT-2, AKT-2 and *MYCN* mRNA and protein expression in control (shCTL), SHMT2 silencing (shSHMT2) and SHMT2 overexpression (pCo-SHMT2) cell lines. (**A**) SHMT2 silencing decreased *AKT-2* mRNA expression by 1.2-fold and SHMT2 overexpression increased *AKT-2* mRNA expression by 1.6-fold in SK-N-AS cells. (**B**) SHMT2 silencing decreased *AKT-2* expression by 2.6-fold in BE(2)-C cells and SHMT2 overexpression increased *AKT-2* mRNA expression by 1.3-fold. *MYCN* mRNA expression was increased by 1.5-fold with SHMT2 silencing in the BE(2)-C cell line compared to control. SHMT2 overexpression decreased *MYCN* mRNA expression by 1.3-fold. (**C**) SHMT2 silencing decreased protein expression of phosphorylated Akt-2 (pAkt-2) and Akt-2 compared to control in SK-N-AS cells (top panel). SHMT2 silencing in SK-N-AS cells markedly increased c-Myc protein expression compared to control, while SHMT2 overexpression minimally increased c-Myc protein expression. Densitometry analysis confirmed SHMT2 silencing decreased pAkt-2 by 1.3-fold, Akt-2 protein expression by 2-fold, and c-Myc protein expression by 2.4-fold compared to control (bottom panel). (**D**) In the BE(2)-C cell lines, N-Myc protein expression was decreased with SHMT2 silencing and overexpression (top panel). SHMT2 silencing did not impact Akt-2 protein expression. SHMT2 overexpression increased pAkt-2 expression, but had no effect on Akt-2 expression. Densitometry analysis, reported as a ratio of each protein band density relative to the density of each ß-actin control band (protein density: ß-actin density), confirmed immunoblotting findings in BE(2)-C cells (bottom panel). SHMT2 silencing resulted in pAkt-2 protein expression 1.6 times lower than control. SHMT2 overexpression increased pAkt-2 protein expression by 1.3-fold. SHMT2 silencing and overexpression had no effect on Akt-2 protein expression. SHMT2 silencing decreased N-Myc protein expression by 1.3-fold and SHMT2 overexpression decreased N-Myc protein expression by 1.8-fold.

In order to assess the potential role of SHMT2 in NB *in vitro*, stable cell lines with SHMT2 silencing and SHMT2 overexpression were created. The *MYCN*-amplified NB cell line, BE(2)-C, and the non-*MYCN*-amplified cell line, SK-N-AS, were used. Both cell lines were transfected with shRNA targeting SHMT2 (shSHMT2) and non-target control shRNA (shCTL), as well as a SHMT2 overexpression plasmid, pCo-SHMT2, to create stably transfected cell lines. Antibiotic selection was performed for two weeks after transfection and successful transfection was confirmed using RT-qPCR (Figure 2A, 2B) and immunoblotting (Figure 2C, 2D).

The effects of SHMT2 silencing and overexpression on *AKT-2* and *MYCN* mRNA expression were evaluated using RT-qPCR. In the non-*MYCN*-amplified cell line, SK-N-AS, SHMT2 silencing decreased *AKT-2* mRNA expression by 1.2-fold, while SHMT2 overexpression increased *AKT-2* mRNA expression by 1.6-fold (Figure 2A). In the *MYCN*-amplified cell line BE(2)-C, SHMT2 silencing decreased *AKT-2* mRNA expression by 2.6-fold, while SHMT2 overexpression increased *AKT-2* mRNA expression by 1.3-fold (Figure 2B). SHMT2 silencing increased *MYCN* mRNA expression by 1.5-fold, while SHMT2 overexpression decreased *MYCN* mRNA expression by 1.3-fold in BE(2)-C cells. The effects of SHMT2 silencing and overexpression on *MYCN* were not assessed in the non-*MYCN*-amplified cell line, SK-N-AS.

Immunoblotting was performed to evaluate the effects of SHMT2 silencing and overexpression on protein expression in both BE(2)-C and SK-N-AS cells. SHMT2 silencing decreased phosphorylated Akt-2 (pAkt-2) expression and SHMT2 overexpression increased pAkt-2 expression in both cell lines (Figure 2C and 2D; top panel). Densitometry analysis showed a 1.3-fold decrease in SK-N-AS pAkt-2 protein expression and 1.6-fold decrease in BE(2)-C pAkt-2 protein expression with SHMT2 silencing (Figure 2C, 2D; bottom panel). SHMT2 overexpression resulted in a 1.3-fold increase in pAkt-2 expression in both cell lines. Neither SHMT2 silencing or overexpression had an effect on total Akt-2 protein expression in the *MYCN*-amplified cell line, BE(2)-C. SHMT2 overexpression had no effect on Akt-2 protein expression in the non-*MYCN*-amplified cell line, SK-N-AS, and SHMT2 silencing resulted in a 2-fold decrease in Akt-2 protein expression. Together these findings suggest SHMT2 affects Akt-2 activation via phosphorylation of Akt-2 in *MYCN*-amplified cells.

The effects of SHMT2 silencing and overexpression on c-Myc protein expression were assessed in the non-*MYCN*-amplified cell line, SK-N-AS. Control cells (shCTL) demonstrated minimal to no c-Myc protein expression. However, SHMT2 silencing increased c-Myc protein expression by 2.4-fold and SHMT2 overexpression increased c-Myc protein expression by 1.6-fold (Figure 2C, bottom panel). SHMT2 silencing decreased N-Myc protein expression in the *MYCN*-amplified cell line, BE(2)-C by 1.3-fold, while SHMT2 overexpression decreased N-Myc protein expression by 1.8-fold (Figure 2D, bottom panel). While SHMT2 silencing decreased N-Myc protein expression, the opposite effect was seen on *MYCN* mRNA expression in the BE(2)-C cell line.

**Figure 3:**
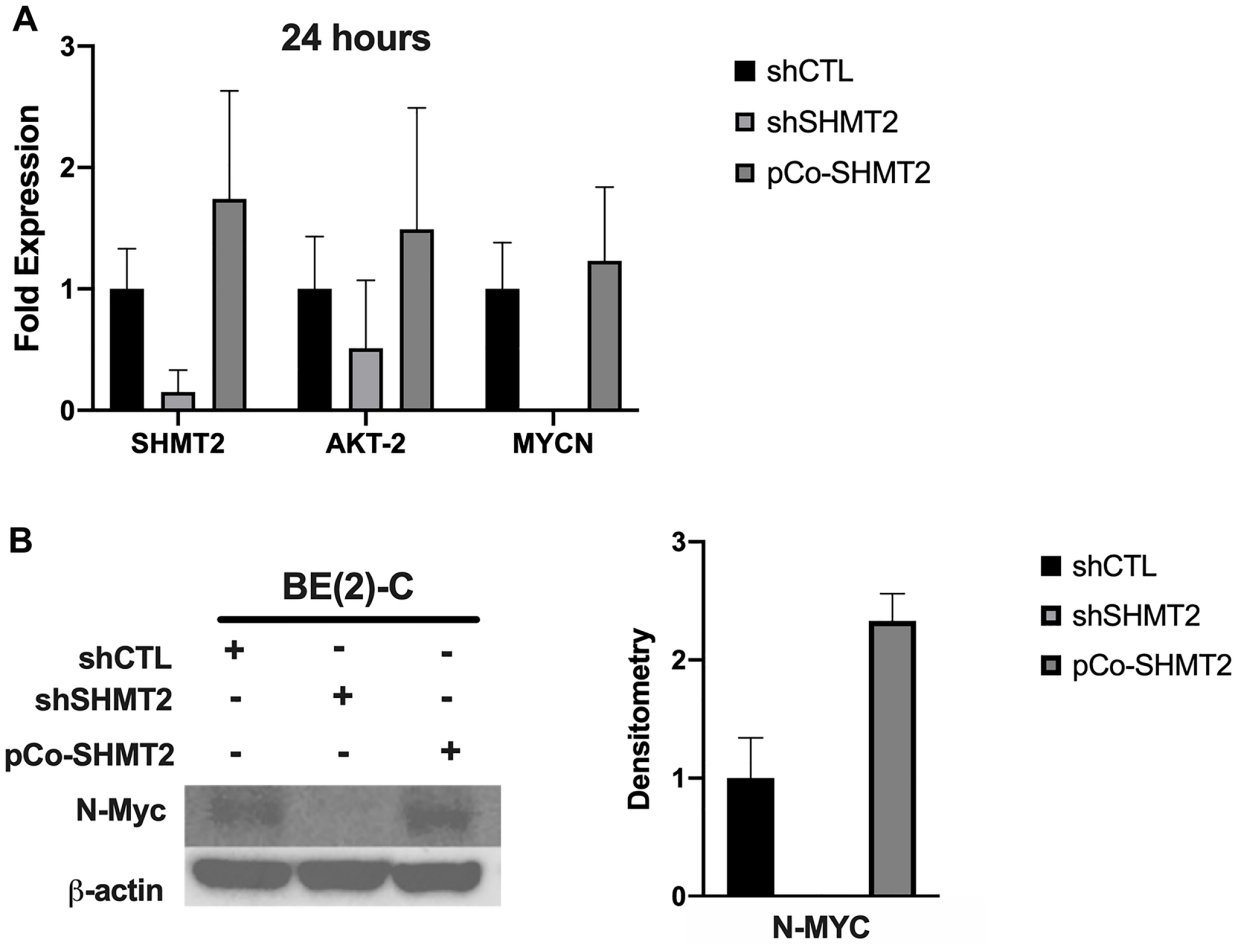
SHMT2 silencing inhibits *MYCN* mRNA and N-Myc protein expression at 24-hours. The effects of SHMT2 silencing and overexpression in BE(2)-C cells were examined 24 hours after plating to control for differences in proliferation rate and cell-cycle. (**A**) SHMT2 silencing decreased *AKT-2* mRNA expression by 2.0-fold and decreased MYCN mRNA expression by 500-fold. SHMT2 overexpression increased *AKT-2* mRNA expression by 1.5-fold and *MYCN* mRNA expression by 1.2-fold. (**B**) SHMT2 silencing completely inhibited N-Myc protein expression and SHMT2 overexpression increased N-Myc protein expression 2.3-fold, based on densitometry analysis, reported as a ratio of each protein band density relative to the density of each β-actin control band (protein density: β-actin density).

Given the incongruent findings between the effects of SHMT2 silencing and overexpression on mRNA and protein expression in the *MYCN*-amplified cell line, BE(2)-C, further analysis was performed. As described in the subsequent findings, SHMT2 overexpression was noted to increase cellular proliferation and SHMT2 silencing decreased cellular proliferation, suggesting shCTL, shSHMT2 and pCo-SHMT2 cells may have been at different cell-cycle phases at 48 hours when mRNA and protein samples were initially collected. In order to account for potential variations in cell cycle, BE(2)-C shCTL, shSHMT2 and pCo-SHMT2 cells were plated on a 6-well plate at 0.25 × 10^6^ cells/well. Protein and mRNA were collected at exactly 24 hours after plating and RT-qPCR and immunoblotting were performed. Findings are summarized in Figure 3. At 24 hours, SHMT2 silencing decreased *AKT-2* mRNA expression by 2.0-fold and nearly eliminated *MYCN* mRNA expression, decreasing it by 500-fold (Figure 3A). SHMT2 overexpression increased *AKT-2* and *MYCN* mRNA expression by 1.5 and 1.2-fold, respectively. Similar findings were seen with immunoblotting. As seen in Figure 3B, SHMT2 silencing completely inhibited N-Myc protein expression at 24-hours. SHMT2 overexpression increased N-Myc protein expression by 2.3-fold at 24 hours. Taken together, these findings suggest SHMT2 silencing inhibits N-Myc expression via decreased activation of Akt-2.

To further elucidate the mechanistic relationship between SHMT2 and *MYCN*-amplification, studies were performed on two additional NB cell lines, SK-N-DZ (*MYCN*-amplified) and SK-N-SH (non-*MYCN* amplified). SHMT2 silencing and overexpression were performed in the same manner as previously described. RT-qPCR and immunoblotting were performed to assess the effects of SHMT2 silencing and overexpression on *AKT-2* and *MYCN* mRNA expression as well as pAkt-2, N-Myc and c-Myc protein expression. SHMT2 silencing resulted in a 1.2-fold decrease in *AKT-2* and 1.1-fold decrease in *MYCN* mRNA expression and SHMT2 overexpression resulted in a 1.2-fold increase in *AKT-2* and a 1.3-fold increase in *MYCN* mRNA expression in the SK-N-DZ cell line (Supplementary Figure 2A). Whereas SHMT2 silencing did not affect *AKT-2* and *MYCN* mRNA expression and SHMT2 overexpression decreased both *AKT-2* and *MYCN* mRNA expression by 1.4-fold and 1.2-fold, respectively, in the SK-N-SH cell line (Supplementary Figure 2D).

Next, the effects of SHMT2 silencing and overexpression on protein expression were examined. SHMT2 silencing decreased N-Myc protein expression by 1.4-fold and pAkt-2 protein expression by 1.1-fold, whereas SHMT2 overexpression increased N-Myc protein expression by 1.2-fold and pAkt-2 protein expression by 1.3-fold in the SK-N-DZ cell line (Supplementary Figure 2B and 2C). In the SK-N-SH cell line, SHMT2 silencing had no effect on N-Myc protein expression and it decreased pAkt-2 protein expression by 1.8 fold. In contrast, SHMT2 overexpression increased N-Myc protein expression by 2.1-fold and decreased pAkt-2 protein expression by 1.1-fold (Supplementary Figure 2F). Interestingly, the SK-N-SH cell line did not demonstrate c-Myc protein expression, as was seen in the additional non-*MYCN* amplified cell line, SK-N-AS (Supplementary Figure 2E). The incongruent effects between *AKT-2* and *MYCN* mRNA expression with SHMT2 silencing suggest SHMT2 silencing does not affect *MYCN* mRNA expression via Akt-2 in the non-*MYCN* amplified cell line, SK-N-SH.

To further evaluate the role of SHMT2 silencing and overexpression on pAkt-2 and N-Myc, the transfected SK-N-AS and BE(2)-C cell lines were treated with the Akt-2 inhibitor, CCT129830, at a dose of 10 μM. Protein samples were collected 24 hours after treatment. The effects of Akt-2 inhibition on N-Myc, c-Myc and pGSK-3α/β (a downstream target of Akt-2) protein expression were assessed (Supplementary Figure 3A and 3D). The effects of SHMT2 overexpression on c-Myc and N-Myc protein expression were markedly less with Akt-2 inhibition. SHMT2 overexpression cells treated with an Akt-2 inhibitor demonstrated a 3.5-fold increase in c-Myc, compared to a 17.3-fold increase in c-Myc without treatment in the SK-N-AS cells (Supplementary Figure 3B). BE(2)-C SHMT2 overexpression cells demonstrated a 1.1-fold increase in N-Myc compared to a 1.3-fold increase in untreated cells (Supplementary Figure 3E). While SHMT2 silencing increased c-Myc protein expression in SK-N-AS cells, it decreased N-Myc protein expression in BE(2)-C cells, as expected. However, treatment with CCT129830 markedly decreased N-Myc protein expression in BE(2)-C cells by 20-fold, compared to an 11-fold decrease in untreated BE(2)-C shSHMT2 cells, suggesting Akt-2 plays an important role in both c-Myc and N-Myc protein expression.

The protein expression of pGSK-3α/β, a downstream target of Akt-2, was evaluated to confirm successful inhibition of Akt-2. As shown in Supplementary Figure 3C, the non-*MYCN* amplified cell line, SK-N-AS, was more susceptible to Akt-2 inhibition, with a 1.4 and 2.9-fold decrease in pGSK-3α/β expression in both SHMT2 silencing and overexpression cells, respectively. Interestingly, treatment with CCT129830 in the BE(2)-C shSHMT2 cells resulted in a 12.5-fold decrease in pGSK-3α/β expression compared to a 2.9-fold decrease with SHMT2 silencing alone, suggesting SHMT2 silencing increased the effectiveness of Akt-2 inhibition (Supplementary Figure 3F). In addition, SHMT2 overexpression resulted in a 1.6-fold increase in pGSK-3α/β expression in untreated cells and a 1.9-fold increase in pGSK-3α/β expression in cells treated with CCT129830, suggesting SHMT2 overexpression cells were able to compensate for Akt-2 inhibition (Supplementary Figure 3F). Taken together, the effects of Akt-2 inhibition on both *MYCN*-amplified and non-*MYCN* amplified NB cells further supports the finding that SHMT2 affects both c-Myc and N-Myc protein expression via Akt-2.

### SHMT2 promotes cellular proliferation, colony formation and migration *in vitro*

**Figure 4:**
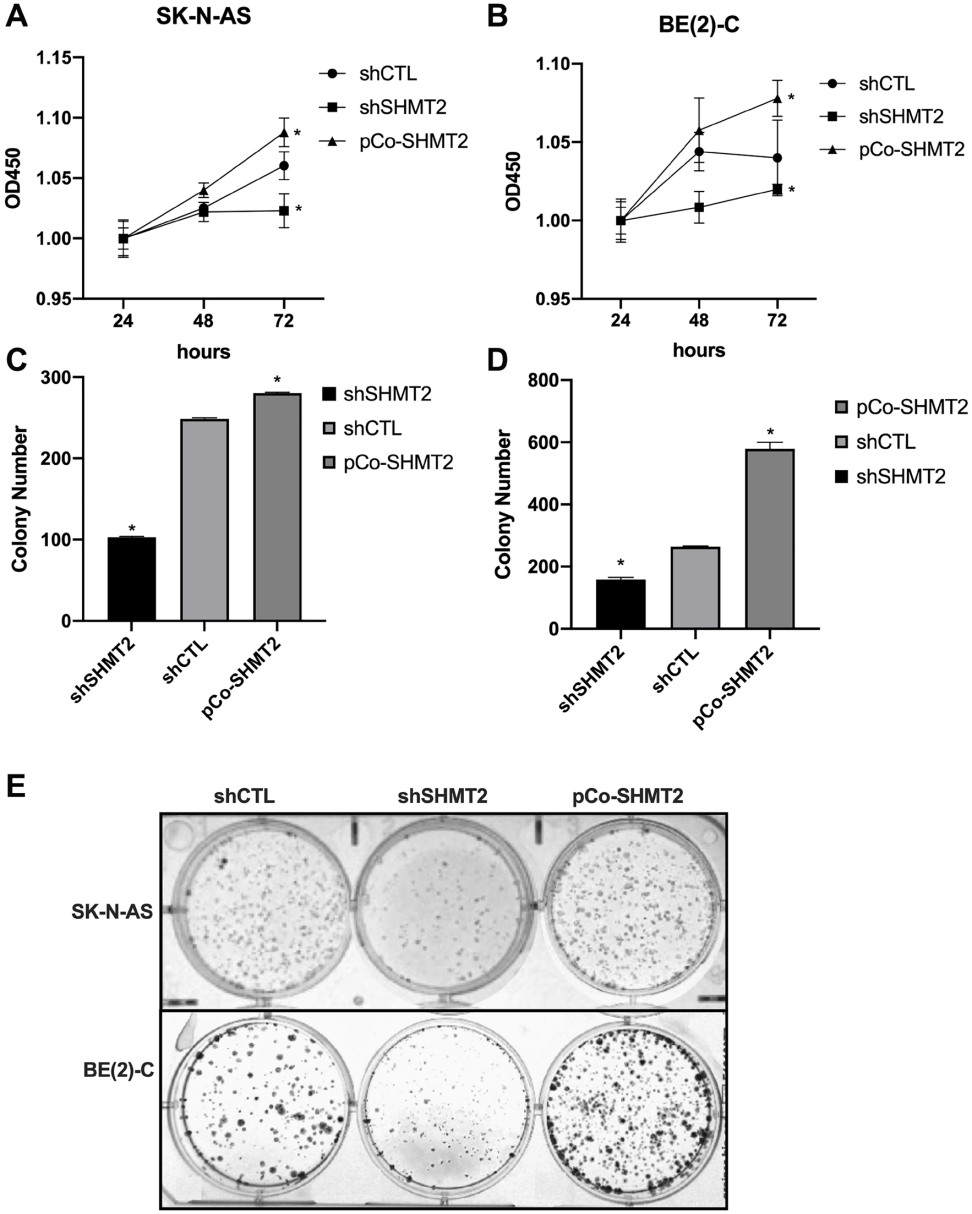
SHMT2 increases cellular proliferation and colony formation *in vitro*. (**A**) SK-N-AS cells were plated on a 96-well plate (1000 cells/well) and cellular proliferation was assessed using CCK-8 assays at 24, 48 and 72 hours after plating. SHMT2 silencing (shSHMT2) decreased cellular proliferation by 1-fold at 72 hours in SK-N-AS cells and SHMT2 overexpression (pCo-SHMT2) increased cellular proliferation by 1-fold at 72 hours. (**B**) BE(2)-C cells plated onto a 96-well plate (500 cells/well) demonstrated increased cellular proliferation by 1-fold at 72 hours with SHMT2 overexpression and decreased cellular proliferation by 1-fold at 72 hours with SHMT2 silencing, compared to control. (**C**) SK-N-AS cells were plated on a 6-well plate at 1000 cells/well for 14 days. Cells were stained with 0.05% crystal violet and colony number was counted. SHMT2 silencing (shSHMT2) resulted in a 2.4-fold decrease in mean colony number compared to control (shCTL). SHMT2 overexpression (pCo-SHMT2) cells demonstrated a 1-fold increase in mean colony number compared to control. (**D**) BE(2)-C cells plated on a 6-well plate at 500 cells/well for 14 days demonstrated a 2.2-fold increase in colony formation with SHMT2 overexpression and 1.7-fold decrease in colony formation with SHMT2 silencing. (**E**) Colony staining with 0.05% crystal violet demonstrates decreased colony formation in SHMT2 silenced cells (shSHMT2) and increased colony formation with SHMT2 overexpression (pCo-SHMT2) compared to control (shCTL). ^*^ *p* < 0.05.

The impact of SHMT2 silencing and overexpression on cellular proliferation was assessed using CCK-8 assays. Cellular proliferation of shCTL, shSHMT2 and pCo-SHMT2 cells in both the SK-N-AS and BE(2)-C cell lines was assessed at 24, 48 and 72 hours. As seen in Figure 4A and 4B, SHMT2 silencing significantly impaired cellular proliferation by 1-fold in both cell lines at 72 hours (*p* < 0.05), while SHMT2 overexpression enhanced cellular proliferation by 1-fold in both cell lines at 72 hours (*p* < 0.05).

Colony counts were performed in both cell lines to assess colony formation in shCTL, shSHMT2 and pCo-SHMT2 cells. SHMT2 silencing significantly decreased colony formation in both SK-N-AS and BE(2)-C cell lines (Figure 4C and 4D). Figure 4E demonstrates colony formation, stained with 0.05% crystal violet, in the SK-N-AS cell lines and BE(2)-C cell lines. SHMT2 silencing impaired colony formation by 2.4-fold in the SK-N-AS cell line and 1.7-fold in the BE(2)-C cell line. Whereas SHMT2 overexpression significantly increased colony formation by 1-fold in the SK-N-AS cell line and 2.2-fold in the BE(2)-C cell line.

**Figure 5:**
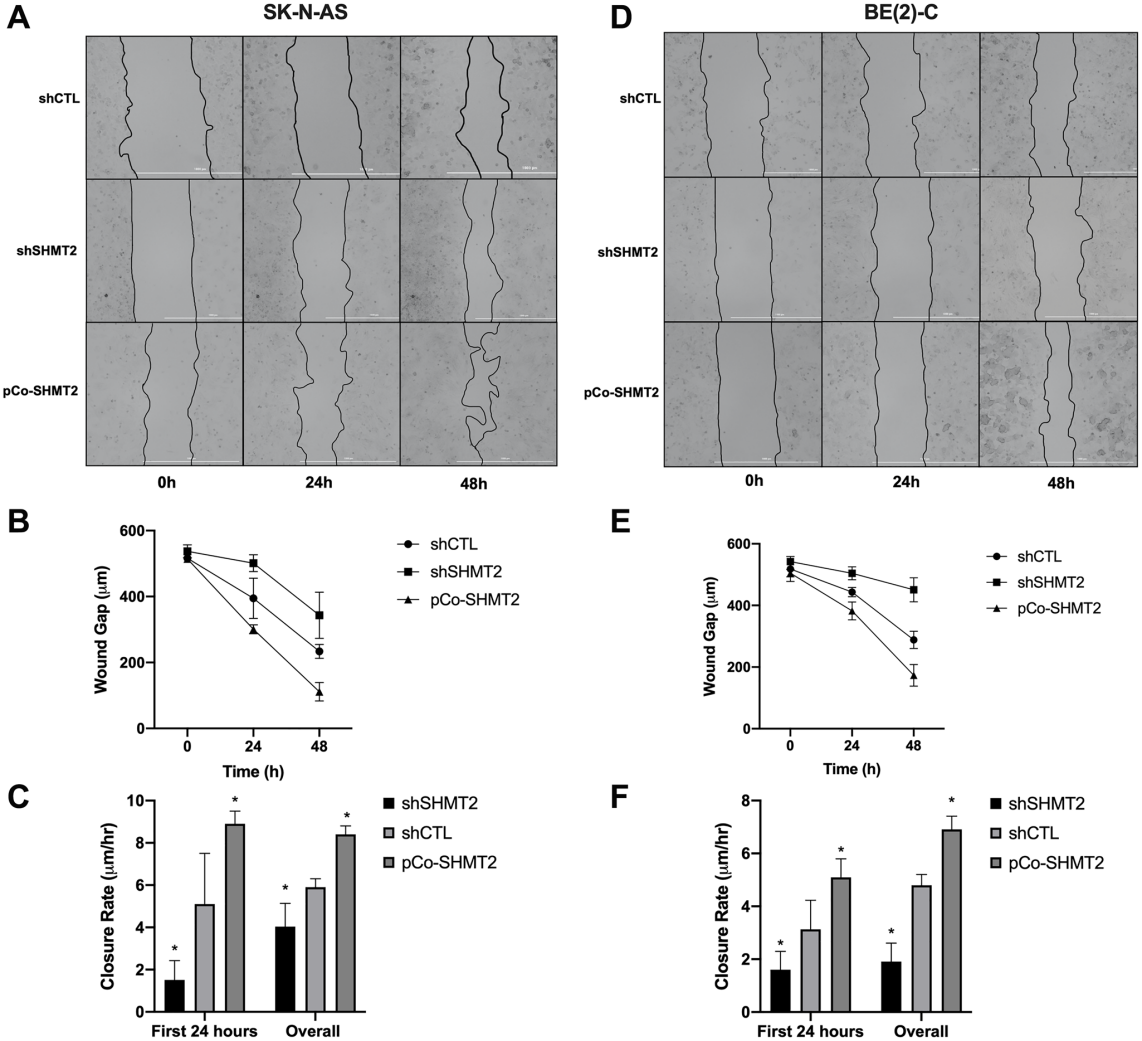
SHMT2 silencing impairs cellular migration in NB. Cells were plated at varying densities (SK-N-AS at 6.0 × 10^5^ cells/mL and BE(2)-C at 2.5 × 10^5^ cells/mL) in 2-well culture inserts (Ibidi). Once 100% confluency was reached, the cell insert was removed, creating an approximately 500 μM gap. Images were obtained using light microscopy at 0, 24 and 48 hours after wound creation. (**A**) SK-N-AS cells at 0, 24 and 48 hours. (**B**) SHMT2 silencing (shSHMT2) increased the average wound gap of SK-N-AS cells and SHMT2 overexpression (pCo-SHMT2) decreased the average wound gap at 0, 24 and 48 hours. (**C**) SHMT2 silencing decreased the first 24-hour mean wound closure rate by 3.4-fold and overall mean wound closure rate by 1.5-fold, while SHMT2 overexpression increased both the first 24 hour and overall mean wound closure rates by 1.7-fold and 1.4-fold, respectively. (**D**) BE(2)-C cells at 0, 24 and 48 hours. (**E**) SHMT2 silencing (shSHMT2) increased the average wound gap of BE(2)-C cells and SHMT2 overexpression (pCo-SHMT2) decreased the average wound gap at 0, 24 and 48 hours. (**F**) SHMT2 silencing decreased the average wound closure rate at day 1 by 2.0-fold and overall by 2.5-fold, while SHMT2 overexpression increased both the day 1 and overall wound closure rates by 1.6-fold and 1.4-fold, respectively. ^*^ *p* < 0.05.

Wound healing assays were performed to assess the effects of SHMT2 overexpression and silencing on cellular migration in both SK-N-AS cells and BE(2)-C cells. Cells were plated in 2-well culture inserts and allowed to reach 100% confluency. The culture inserts were then removed, creating an approximately 500 μm wound. The wound gaps were measured at 0, 24 and 48 hours after wound creation. Images of SK-N-AS cells and BE(2)-C cells undergoing wound healing were captured with light microscopy and are shown in Figure 5A and 5D, respectively. In SK-N-AS cells, SHMT2 silencing resulted in a bigger wound gap compared to control (Figure 5B). SHMT2 silencing also decreased the wound closure rate in the first 24 hours (5.1 μm/hr (shCTL) vs 1.5 μm/hr (shSHMT2), *p* < 0.05) and overall (5.9 μm/hr (shCTL) vs 4.0 μm/hr (shSHMT2), *p* < 0.05), compared to control (Figure 5C). SHMT2 overexpression led to a decreased wound gap at 24 and 48 hours (Figure 5B) and an increased wound closure rate in the first 24 hours (5.1 μm/hr (shCTL) vs 8.9 μm/hr (pCo-SHMT2), *p* < 0.05) and overall (5.9 μm/hr (shCTL) vs 8.4 μm/hr (pCo-SHMT2), *p* < 0.05), compared to control (Figure 5C). Similar findings were seen in the BE(2)-C cell line. SHMT2 silencing resulted in an increased wound gap at 24 and 48 hours (Figure 5E), as well as a decreased wound closure rate at 24 hours (3.13 μm/hr (shCTL) vs 1.6 μm/hr (shSHMT2), *p* < 0.05) and overall (4.8 μm/hr (shCTL) vs 1.9 μm/hr (shSHMT2), *p* < 0.05), compared to control (Figure 5F). SHMT2 overexpression resulted in a decreased wound gap at both 48 and 72 hours, compared to control (Figure 5E) and a significant increase in the 24-hour wound closure rates (3.1 μm/hr (shCTL) vs 5.1 μm/hr (pCo-SHMT2), *p* < 0.05) and overall wound closure rates (4.8 μm/hr (shCTL) vs 6.9 μm/hr (pCo-SHMT2), *p* < 0.05), compared to control (Figure 5F). Overall, these findings indicate that SHMT2 silencing impairs cellular migration, whereas SHMT2 overexpression enhances cellular migration.

To evaluate the potential role of c-Myc expression on NB tumor function, the effects of SHMT2 silencing and overexpression on colony formation in the non-*MYCN* amplified cell line, SK-N-SH, were examined. Cells were plated at 1000 cells/well on a 6-well plate in triplicate and colony formation was assessed at 7 days. There were fewer colonies seen with SHMT2 silencing and larger colonies seen with SHMT2 overexpression. However, there was no significant difference in colony formation between shCTL and shSHMT2 cells, but there was a significant decrease in colony formation in pCo-SHMT2 cells compared to shCTL (shCTL 280.7 ± 36 colonies vs 231.8 ± 10 colonies, *p* = 0.001) (Supplementary Figure 4A). However, this is likely due to pCo-SHMT2 cells overgrowing the plate and forming larger colonies compared to shCTL and shSHMT2 cells, as seen in the representative images. (Supplementary Figure 4B). These findings suggest SHMT2 silencing and overexpression affected colony formation via N-Myc or c-Myc and that there was no functional effect on the non *MYCN*-amplified cell line, SK-N-SH, which lacks c-Myc.

### Metastatic NB cells demonstrate increased SHMT2 mRNA and protein expression

**Figure 6:**
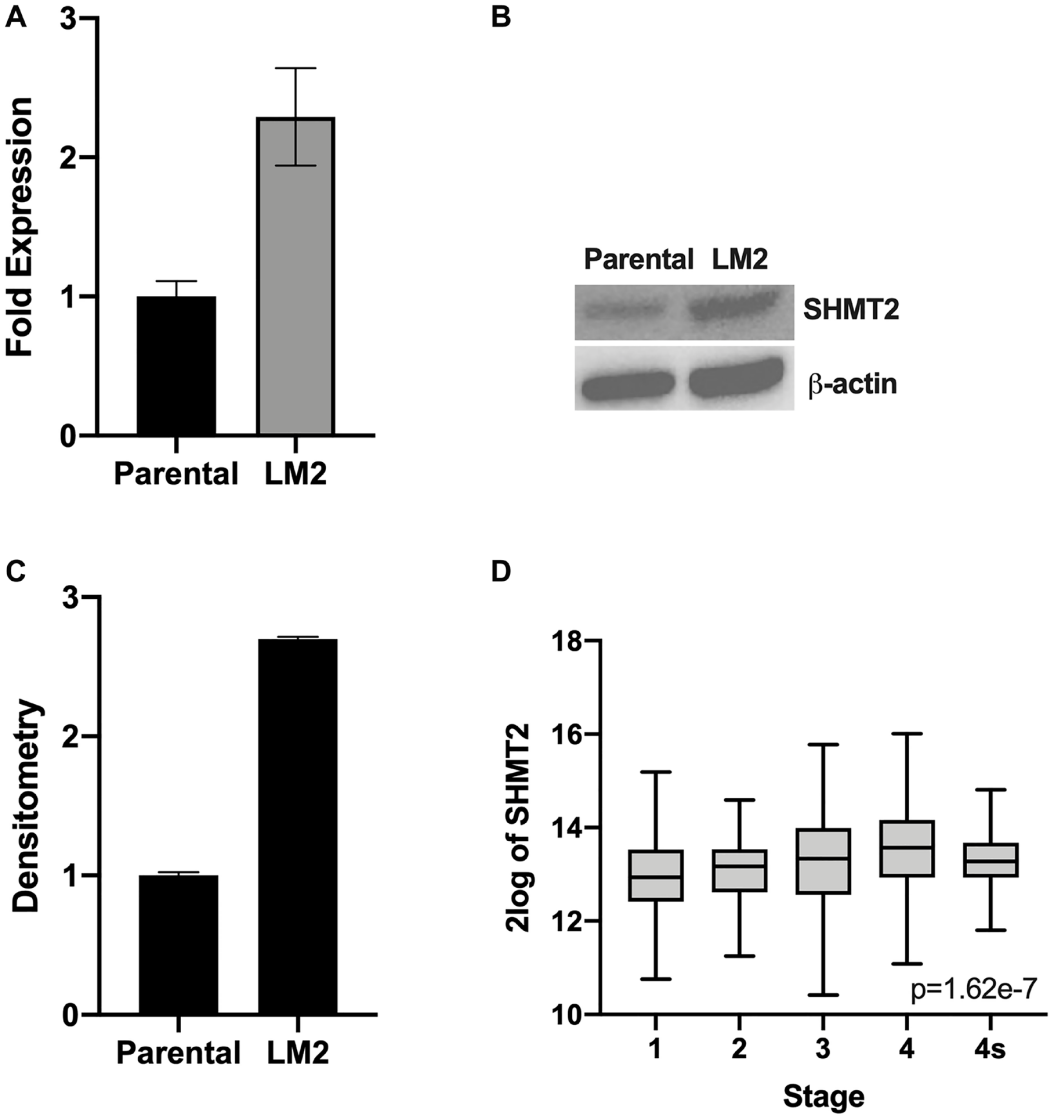
SHMT2 expression is increased in metastatic NB cells. RT-qPCR and immunoblotting were used to assess SHMT2 mRNA and protein expression, respectively, in the highly aggressive pro-metastatic cell line, LM2, and the BE(2)-C parental cell line. (**A**) *SHMT2* mRNA expression is markedly increased in the metastatic cell line LM2 compared to parental cells. (**B**) SHMT2 protein expression is increased in LM2 compared to parental cells. (**C**) Densitometry analysis, reported as a ratio of each protein band density relative to the density of each β-actin control band (protein density: β-actin density), demonstrates SHMT2 protein expression is 2.7 times higher in metastatic LM2 cells compared to the BE(2)-C parent cells. (**D**) R2 genomics database analysis of *SHMT2* gene expression based on disease stage demonstrates increasing *SHMT2* expression with increasing disease stage in neuroblastoma. The highest SHMT2 expression was seen in patients with stage 4, metastatic disease.

To further evaluate the role of SHMT2 on NB metastatic potential, the LM2 cell line was evaluated. The LM2 cell line, which was established in our laboratory previously [13], is a highly aggressive NB cell line with propensity to metastasize that was obtained from BE(2)-C cell liver metastases after splenic injection for two cycles. In order to assess for SHMT2 mRNA and protein expression, RT-qPCR and immunoblotting were performed. Figure 6A demonstrates a 2.3-fold increase in SHMT2 mRNA expression in the metastatic cell line, LM2, compared to parental BE(2)-C cells. Immunoblotting also demonstrated a 2.7-fold increase in SHMT2 protein expression in the LM2 cells compared to parental BE(2)-C cells (Figure 6B and 6C). This result indicated that SHMT2 may affect the tumor cell spread *in vitro*. Therefore, the R2 genomics analysis platform was used to analyze *SHMT2* expression in NB patients based on stage. Figure 6D demonstrates increasing *SHMT2* expression with increasing stage (excluding 4S), with the highest *SHMT2* expression seen in patients with stage 4, metastatic disease. Together these results indicate SHMT2 plays an important role in NB metastasis *in vitro* and that *SHMT2* expression is increased in patients with metastatic NB.

**Figure 7:**
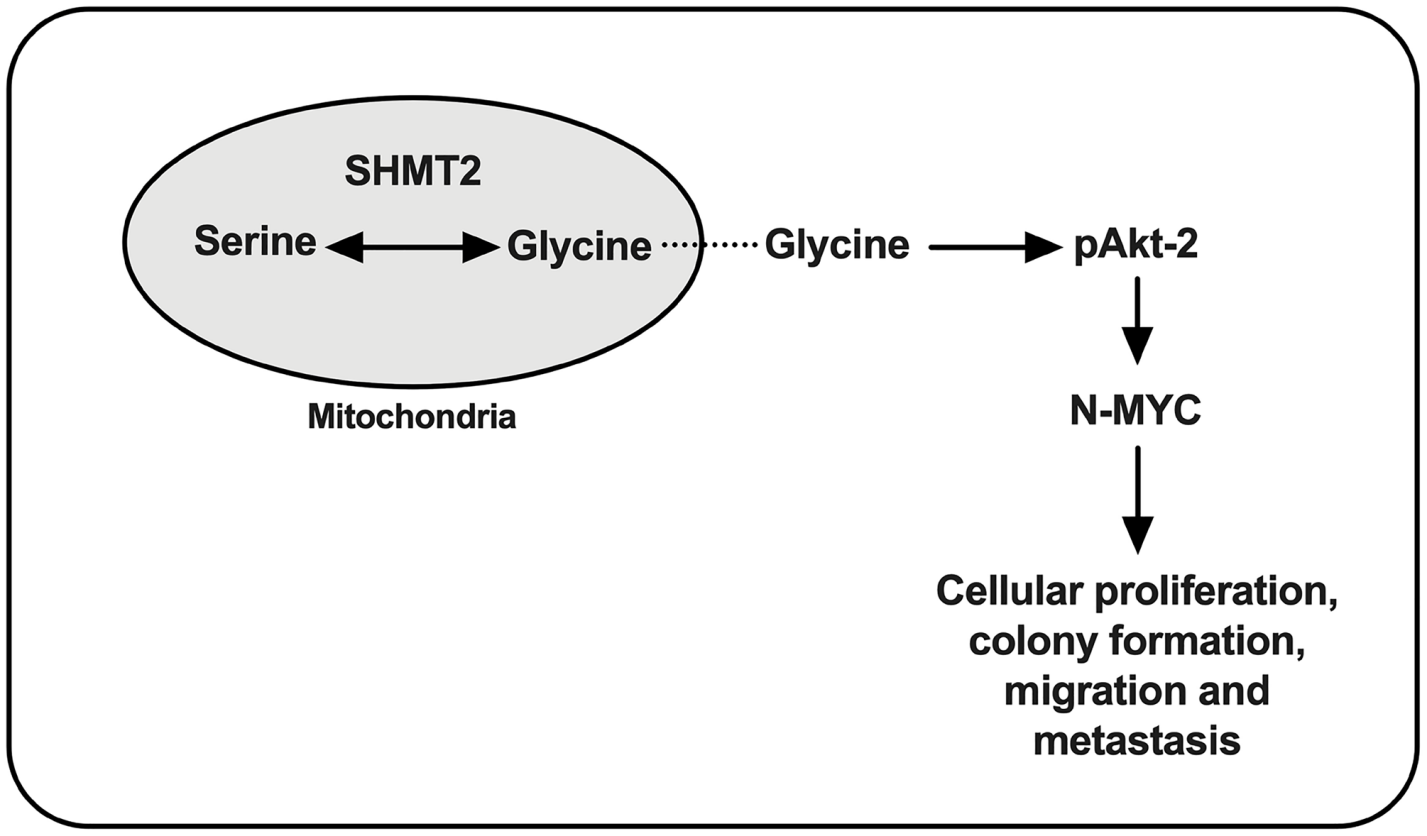
Proposed cellular pathway of SHMT2 in NB tumorigenesis. Serine is metabolized to glycine via SHMT2 and leads to phosphorylation of Akt-2 (pAkt-2), which increases N-Myc protein stabilization and contributes to NB tumorigenesis.

In summary, SHMT2 plays an important role in NB tumorigenesis. Based on the findings above, SHMT2 increases N-Myc mRNA and protein expression via phosphorylation/activation of Akt-2, leading to increased cellular proliferation, colony formation, migration and metastasis (Figure 7).

## DISCUSSION

NB is the most common pediatric, extracranial, solid tumor, accounting for approximately 15% of pediatric cancer deaths [1]. Poor prognostic factors in children with NB include: age greater than 18 months at the time of diagnosis, unfavorable histology, increased vascularization and *MYCN* gene amplification. A common deregulated metabolism pathway is the MYC gain-of-function pathway. Increased MYC gene expression affects several aspects of metabolism, including: glycolysis, mitochondrial function and serine metabolism. Serine metabolism is affected in a MYC-dependent manner by the mitochondrial enzyme SHMT2 [4]. Upregulation of SHMT2 is found in several cancers and is associated with increased tumor aggressiveness. SHMT2 expression is an independent predictor of prognosis in glioma, breast and lung cancer [8, 9, 14, 15]. The PI3K/Akt pathway regulates angiogenesis by stabilizing N-myc and is a commonly deregulated pathway in high-risk NB [11]. In addition, increased expression of SHMT2 in hepatocytes has been shown to lead to physiologic Akt activation via PI3K [12]. Given the common pathway, we hypothesized that SHMT2 plays a critical role in NB tumorigenesis and metastasis via the PI3K/Akt pathway.

In the present study, we found that increased *SHMT2* gene expression was associated with *MYCN*-amplification and a decrease in overall survival, in NB patients. These findings are similar to previous studies which have demonstrated an upregulation of SHMT2 in several cancers such as lymphoma, glioma, cholangiocarcinoma, breast cancer, gastric cancer, lung adenocarcinoma and colorectal cancer [6, 7, 14, 16]. In addition, we found that SHMT2 mRNA and protein expression were increased in the aggressive *MYCN*-amplified cell lines BE(2)-C and SK-N-DZ, compared to the non-*MYCN*-amplified cell lines, SK-N-AS and SK-N-SH. These findings are similar to a previous study which found that SHMT2 was upregulated in *MYCN*-amplified NB cells [10]. Several studies have demonstrated an association between SHMT2 expression and tumor aggressiveness, suggesting that SHMT2 is an independent predictor of prognosis in glioma, breast and lung cancer [8, 9, 14, 15]. In addition, many related genes involved in serine metabolism have been associated with advanced stage disease and decreased overall survival in NB [17].

We have previously demonstrated that activation of Akt-2 specifically, is associated with NB tumor development, progression and metastasis [18]. A physiologic link between serine/glycine metabolism, SHMT2 and Akt has been shown in hepatocytes. However, to the best of our knowledge, this is not only the first investigation into the relationship between PI3K/Akt and SHMT2 in NB, but in all cancer studies [12]. In our study, we found that SHMT2 silencing decreased *AKT-2* mRNA expression and pAkt-2 activity. In addition, SHMT2 silencing completely inhibited N-Myc protein expression at 24 hours and decreased *MYCN* mRNA expression by 500-fold in BE(2)-C cells, suggesting SHMT2 regulates N-Myc via decreased activation of Akt-2. Our studies were performed on two additional cell lines, the non-*MYCN* amplified cell line, SK-N-SH, and the *MYCN*-amplified cell line, SK-N-DZ. Similar to BE(2)-C cells, the *MYCN*-amplified SK-N-DZ cells demonstrated decreased pAkt-2 and N-Myc protein expression with SHMT2 silencing and increased pAkt-2 and N-Myc protein expression with SHMT2 overexpression. However, there was no association between SHMT2 overexpression or silencing on pAkt-2 and N-Myc expression in the SK-N-SH cell line. Notably, the non-MYCN amplified cell line, SK-N-SH, which is known to have a single copy of *MYCN* [19], demonstrated N-Myc protein expression, but revealed minimal to no c-Myc protein expression. These findings are similar to previous studies which demonstrated a key role in the relationship between SHMT2 and MYC on cellular proliferation in other cancers, such as glioma [7, 10].

Reprogrammed metabolism allows for pathologic cell survival. Some metabolic pathways are less important in solitary tumors but essential for metastasis [4]. In order to metastasize, cells must endure nutrient-poor and hypoxic conditions. SHMT2 has been shown to be upregulated in hypoxia and plays a key role in proliferation in areas of ischemia in other cancers, such as gliomas and breast cancer [6, 10, 15]. The ability to adapt to hypoxic environments suggests NB cells with increased SHMT2 expression may be more readily able to metastasize given their ability to endure hypoxic environments. As expected, in the present study we found that SHMT2 silencing significantly impaired cellular proliferation, colony formation and cellular migration, while SHMT2 overexpression enhanced cellular proliferation, colony formation and cellular migration *in vitro.*

Although RT-qPCR and immunoblotting suggest the importance of SHMT2 in *MYCN* mRNA and N-Myc protein expression, SHMT2 silencing and overexpression impacted cellular function in both the *MYCN*-amplified cell line BE(2)-C and the non-*MYCN*-amplified cell line, SK-N-AS. This may be due to induction of c-Myc in the SK-N-AS cell line. In addition, the effects of cellular proliferation and colony formation with SHMT2 overexpression were more pronounced in the *MYCN*- amplified cell line, BE(2)-C, likely due to an increased *MYCN* level at baseline compared to the non-MYCN-amplified cell line, SK-N-AS. However, as previously discussed, SHMT2 silencing decreased colony formation and SHMT2 overexpression increased colony formation and c-Myc protein expression in the non-*MYCN* amplified cell line, SK-N-AS. Whereas, SHMT2 silencing and overexpression in the non-*MYCN* amplified cell line, SK-N-SH, which does not express c-Myc, did not significantly affect colony formation. Taken together, these results suggest SHMT2 affects cellular function via N-Myc or c-Myc protein expression, via pAkt-2, and that neither SHMT2 silencing or overexpression impacted cellular function in the non-*MYCN* amplified cell line, SK-N-SH. Moreover, while the effects of SHMT2 silencing on cellular proliferation, migration and colony formation may be related to decreased *MYCN*/N-Myc expression, different mechanisms such as induction of differentiation, may be related to SHMT2 and will be evaluated in future studies.

Given the effects of SHMT2 overexpression on cellular behavior, particularly colony formation and cellular migration, we sought to evaluate the role of SHMT2 in NB metastasis. We found increased SHMT2 mRNA and protein expression in the metastatic, highly aggressive cell line, LM2, compared to parental control. In addition, analysis of *SHMT2* expression with relation to NB disease stage revealed increasing *SHMT2* gene expression with increasing stage, with the highest SHMT2 expression in metastatic, stage 4 NB. These findings are concordant with a previous study which found increased SHMT2 expression in metastatic breast cancer tissue compared to primary tumor tissue [15].

In conclusion, SHMT2 expression is associated with poor overall survival and high-risk, *MYCN*-amplified NB. At the cellular level, SHMT2 silencing regulates N-Myc via decreased activation of Akt-2 and plays a key role in NB cellular proliferation, colony formation and cellular migration *in vitro.* Further studies are needed to evaluate the role of SHMT2 in NB *in vivo*. Elucidating the underlying pathophysiology and mechanisms of SHMT2 on reprogrammed metabolism is key to furthering our understanding of high-risk NB behavior and developing potential therapeutic targets in order to exploit the dependence of high-risk NB on altered serine metabolism. In addition, given SHMT2 is upregulated in several cancers, elucidating the mechanism underlying SHMT2 in NB will aid the development of potential therapies with broad applications.

## MATERIALS AND METHODS

### Antibodies and reagents

The primary antibodies SHMT2 and β-actin were obtained from Sigma-Aldrich (St. Louis, MO, USA). The primary antibodies N-Myc, c-Myc, pGSK-3α/β, pAkt-2 (ser474) and Akt-2 were obtained from Cell Signaling Technology (Danvers, MA, USA). Secondary goat anti-rabbit antibodies were obtained from Santa Cruz Biotechnology, Inc. (Santa Cruz, CA, USA). The plasmid pCo-SHMT2 (pMXS-IRES-Blast coSHMT2) was a gift from Richard Possemato (Addgene plasmid # 106299; http://n2t.net/ addgene:106299; RRID:Addgene_106299) [20]. The shRNA targeting SHMT2 (shSHMT2) and the control vector SHC002 (shCTL) were purchased from Sigma-Aldrich. Blasticidin and puromycin were purchased from Invivogen (San Diego, CA, USA).

### Cell culture

The human NB cell lines BE(2)-C, SK-N-DZ, SK-N-SH and SK-N-AS were purchased from the American Type Culture Collection (Manassas, VA, USA). Cells were maintained in RPMI 1640 with 10% Fetal Bovine Serum (FBS) at 37°C in a humidified atmosphere consisting of 5% CO_2_ and 95% air.

### Plasmids, shRNA and transfections

Human NB cells were transfected with plasmids, shRNA, shCTL and pCo-SHMT2, using Lipofectamine 3000 (Life Technologies) according to the manufacturer’s instructions. Antibiotic selection was performed on stably-transfected shSHMT2 and shCTL cells with puromycin (10 mg/mL) at 0.5 μg/mL for SK-N-AS cells and 2.5 μg/mL for BE(2)-C cells for two weeks. Antibiotic selection for stably-transfected pCo-SHMT2 cells was performed with blasticidin (10 mg/mL) at 16.0 μg/mL for SK-N-AS cells and 8.0 μg/mL for BE(2)-C cells. The antibiotic selection doses for the SK-N-AS cells and BE(2)-C cells were determined by performing antibiotic kill curves with increasing doses of puromycin and blasticidin.

### SHMT2 gene expression analysis

The R2: Genomics Analysis and Visualization Platform (http://r2.amc.nl, http://r2platform.com) and the Kocak - 649 - custom - ag44kcwolf public NB database were used to create Kaplan-Meier survival curves in order to assess the relationship between *SHMT2* expression and survival in NB patients. The R2 genomics database determines high versus low expression by performing a KaplanScan, a log rank test on increasing gene expression and defines a “cut-off” value of high versus low expression based on the value with the most significant expression value for performing survival analysis. Additional box and whisker plots of the relationship between *SHMT2* expression and *MYCN* amplification, as well as NB disease stage, were also created.

### Akt-2 inhibition

The Akt-2 inhibitor, CCT129830, was used to assess the effects of Akt-2 inhibition on downstream targets such as N-Myc, c-Myc and pGSK-3α/β [21]. BE(2)-C and SK-N-AS cells were plated at 0.25 × 10^6^ and 0.5 × 10^6^ cells/well, respectively, in a 6-well plate. Cells were permitted to attach overnight and were then treated with control media or 10 μM of CCT129830, based on IC_50_ dosing calculated in a previous study [22]. Lysate was collected after 24 hours of treatment and immunoblotting was performed.

### Cell viability assay

To perform the cell viability assays, cells were seeded onto 96-well plates at equivalent densities (BE(2)-C cells at 500 cells/per well and SK-N-AS cells at 1000 cells/well) in RPMI culture medium with 10% FBS. Cell viability was measured using Cell Counting Kit-8 (CCK-8) colorimetric assay (Dojindo Molecular Technologies, Inc., Rockville, MD, USA) at 24, 48, 72 and 96 hours after seeding.

### Clonogenic assay

Cells were plated at clonal density (BE(2)-C cells at 500 cells/well and SK-N-SH/SK-N-AS cells at 1000 cells/well) on a 6-well plate, in triplicate. Cells were permitted to attach and grow for either a 7 or 14-day period. Colonies were stained with 0.05% crystal violet, photographed and counted.

### Wound healing assay

Cellular migration was assessed using a wound healing assay. Cells were plated onto 6-well plate using 2-cell culture inserts (Ibidi USA, Inc., Fitchburg, WI) with a predetermined gap of 500 μm at a density of 2.5 × 10^5^ cells/mL for BE(2)-C cells and 6.0 × 10^5^ cells/mL for SK-N-AS cells. The cells were allowed to reach 100% confluency at 24 hours and the culture insert was removed, creating an approximately 500 μm gap. Cells were washed with PBS and grown in 2 mL of RPMI with 10% FBS. The wound gap was photographed, and the distance measured in 3 separate locations at 0, 24 and 48 hours after culture insert was removed. The average wound gap was calculated for each cell line at 0, 24 and 48 hours. Both an overall and 24-hour wound closure rate were calculated for each cell line.

### RNA isolation and qPCR with reverse transcription

Total RNA was isolated and purified using the RNeasy isolation kit (Qiagen, Germantown, MD, USA) with DNase digestion. The High-Capacity cDNA Reverse Transcription Kit (Applied Biosystems, Carlsbad, CA, USA) was used to synthesize cDNA. RT-qPCR and data collection were performed on a CFX96 instrument (Bio-Rad). Data were normalized to an endogenous control, *GAPDH*. Amplification was performed for 40 cycles of 30 s at 95°C, 30 s at 55°C, and 40 s at 72°C. Primers of *GAPDH* have been described previously [23]. *SHMT2* target primers are (forward 5′-CGAGTTGCGATGCTGTACTT-3′; reverse 5′-CTGCGTTGCTGTGCTGAG-3′). Additional target primers are *MYCN* (forward 5′-GCTTCTACCCGGACGAAGATG-3′; reverse 5′-CAG CTCGTTCTCAAGCAGCAT-3′) and *AKT-2* (forward 5′-AAAGTCATCCTGGTGCG-3′; reverse 5′-GGGTGCCTGGTGTTCTG-3′).

### Immunoblotting

Cells were collected using cell lysis buffer and denatured samples were prepared for immunoblotting. Equal amounts of protein were loaded and separated by NuPAGE 4–12% Bis-Tris gel, followed by transfer onto PVDF membranes (Bio-Rad, Hercules, CA, USA). Membranes were blocked with 5% nonfat milk in TBS-T for 1 hour at room temperature (RT). The blots were then incubated with antibodies against the human target proteins by using rabbit or mouse anti-human antibodies (1:500–2000 dilution) overnight at 4°C. Membranes were incubated with anti-rabbit or anti-mouse secondary antibodies conjugated with HRP for 1 hour at RT and visualized using an enhanced chemiluminescence detection system (PerkinElmer, Waltham, MA, USA). Densitometry was used to assess quantitative protein expression using ImageJ software (Rasband, W.S., ImageJ, U. S. National Institutes of Health, Bethesda, Maryland, USA, https://imagej.nih.gov/ij/, 1997–2018).

### Statistical analysis and experimental analysis

All experiments were repeated in triplicate. The scoring index and relative expression values were expressed as mean ± SEM or SD; statistical analyses were performed using student *t*-tests and analyses of variance for comparisons between the groups. A *p* value of < 0.05 was considered significant.

## SUPPLEMENTARY MATERIALS



## Abbreviations

NB: neuroblastoma
SHMT2: serine hydroxymethyltransferase 2
PI3K: phosphatidylinositol 3-kinase
OS: overall survival
RT-qPCR: Reverse Transcription-quantitative polymerase chain reaction
pAkt-2: phosphorylated Akt-2
FBS: fetal bovine serum
