# Supplementary material for: Induction of serine hydroxymethyltransferase 2 promotes tumorigenesis and metastasis in neuroblastoma
Source: Oncotarget. 2022 Jan 6;13:32–45. doi: 10.18632/oncotarget.28168 (PMC8735882; doi:10.18632/oncotarget.28168)
Supplement: Supplementary file 1 [file oncotarget-13-28168-s001.pdf]

# Induction of serine hydroxymethyltransferase 2 promotes tumorigenesis and metastasis in neuroblastoma

## SUPPLEMENTARY MATERIALS

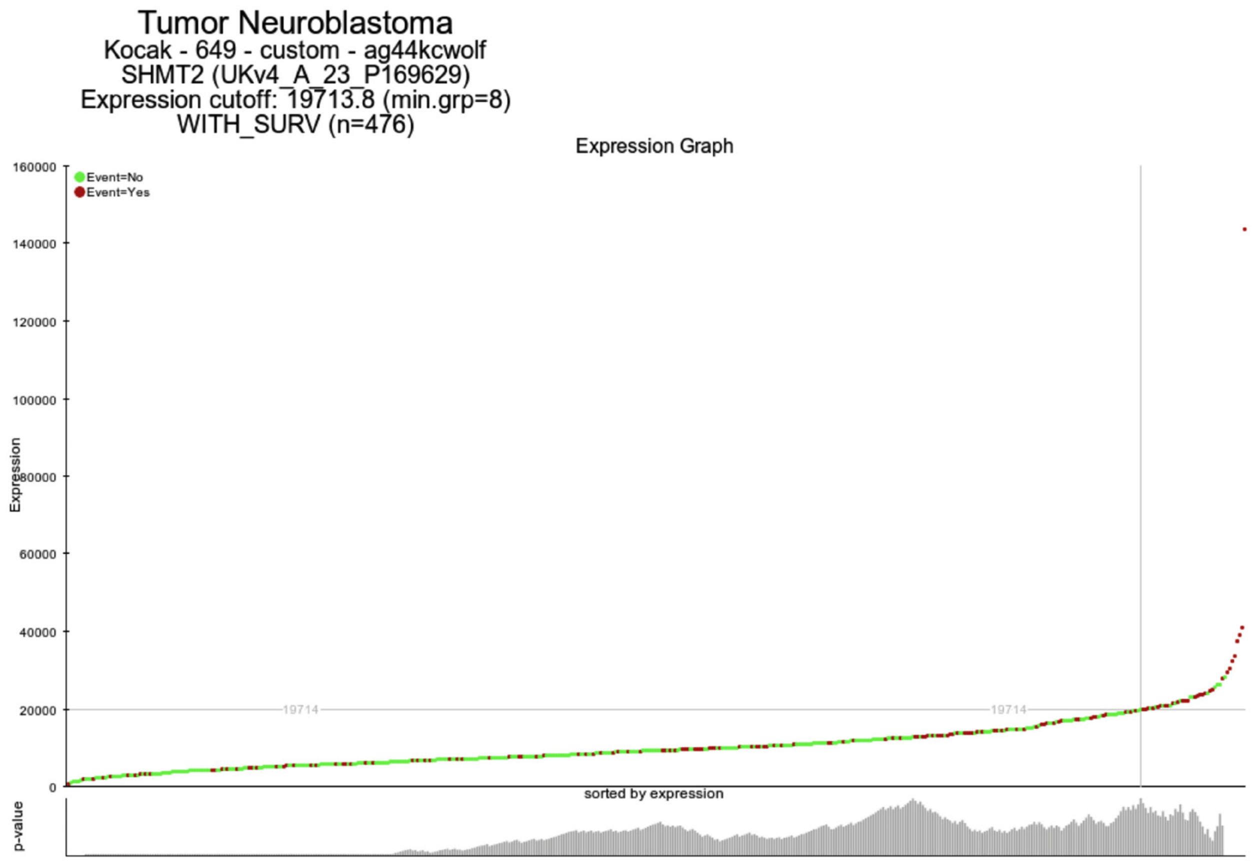


**Supplementary Figure 1:** Expression graph of SHMT2 gene expression level with corresponding *p*- values, demonstrating the optimal gene expression cut-off value for high versus low SHMT2 expression in the Kocak database was 19713.8.

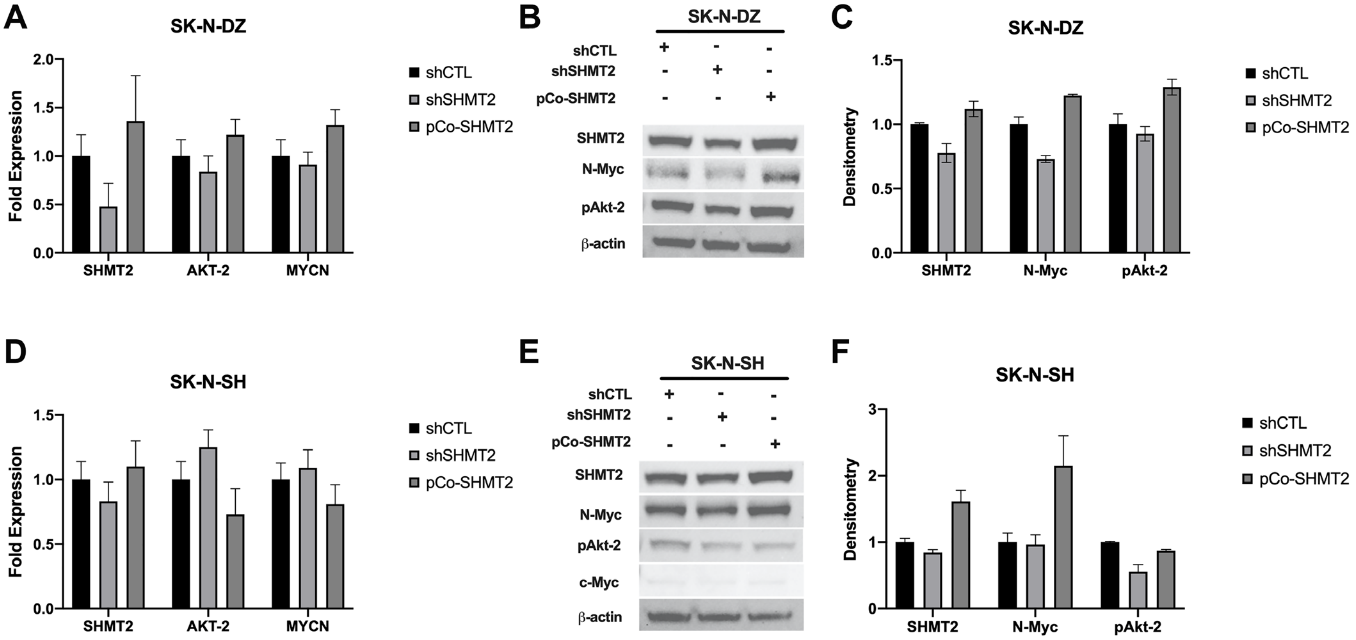


**Supplementary Figure 2: SHMT2 silencing and overexpression in the *MYCN*-amplified cell line, SK-N-DZ, affected AKT-2/pAkt-2 and *MYCN*/N-Myc mRNA and protein expression, whereas, SHMT2 silencing and overexpression had no effect on AKT-2/pAkt-2 and *MYCN*/N-Myc mRNA and protein expression in the non-*MYCN* amplified cell line, SK-N-SH.** (**A**) SHMT2 silencing in SK-N-DZ cells decreased AKT-2 and *MYCN* mRNA expression by 1.2-fold and 1.1-fold, respectively. SHMT2 overexpression increased AKT-2 and *MYCN* mRNA expression by 1.2-fold and 1.3-fold, respectively. (**B**) SHMT2 silencing decreased pAkt-2 and N-Myc protein expression, whereas SHMT2 overexpression increased pAkt-2 and N-Myc protein expression in SK-N-DZ cells. (**C**) Densitometry analysis, reported as a ratio of each protein band density relative to the density of each β-actin control band (protein density: β-actin density), demonstrated a 1.1-fold and 1.4-fold decrease in pAkt-2 and N-Myc protein expression, respectively, with SHMT2 silencing in SK-N-DZ cells. SHMT2 overexpression resulted in a 1.3 and 1.2-fold increase in pAkt-2 and N-Myc protein expression, respectively. (**D**) SHMT2 silencing and overexpression in the non-MYCN amplified cell line, SK-N-DZ. SHMT2 silencing increased AKT-2 and *MYCN* mRNA expression by 1.3 and 1.1-fold, respectively. SHMT2 overexpression decreased AKT-2 and *MYCN* mRNA expression by 1.4 and 1.2-fold, respectively. (**E**) SHMT2 silencing had minimal to no effect on N-Myc protein expression and decreased pAkt-2 protein expression. SHMT2 overexpression increased pAkt-2 and N-Myc protein expression. (**F**) Densitometry analysis, reported as a ratio of each protein band density relative to the density of each β-actin control band (protein density: β-actin density), demonstrated no change in N-Myc and a 1.8-fold decrease in pAkt-2 protein expression with SHMT2 silencing. SHMT2 overexpression increased N-Myc and pAkt-2 protein expression by 2.1 and 1.1-fold, respectively.

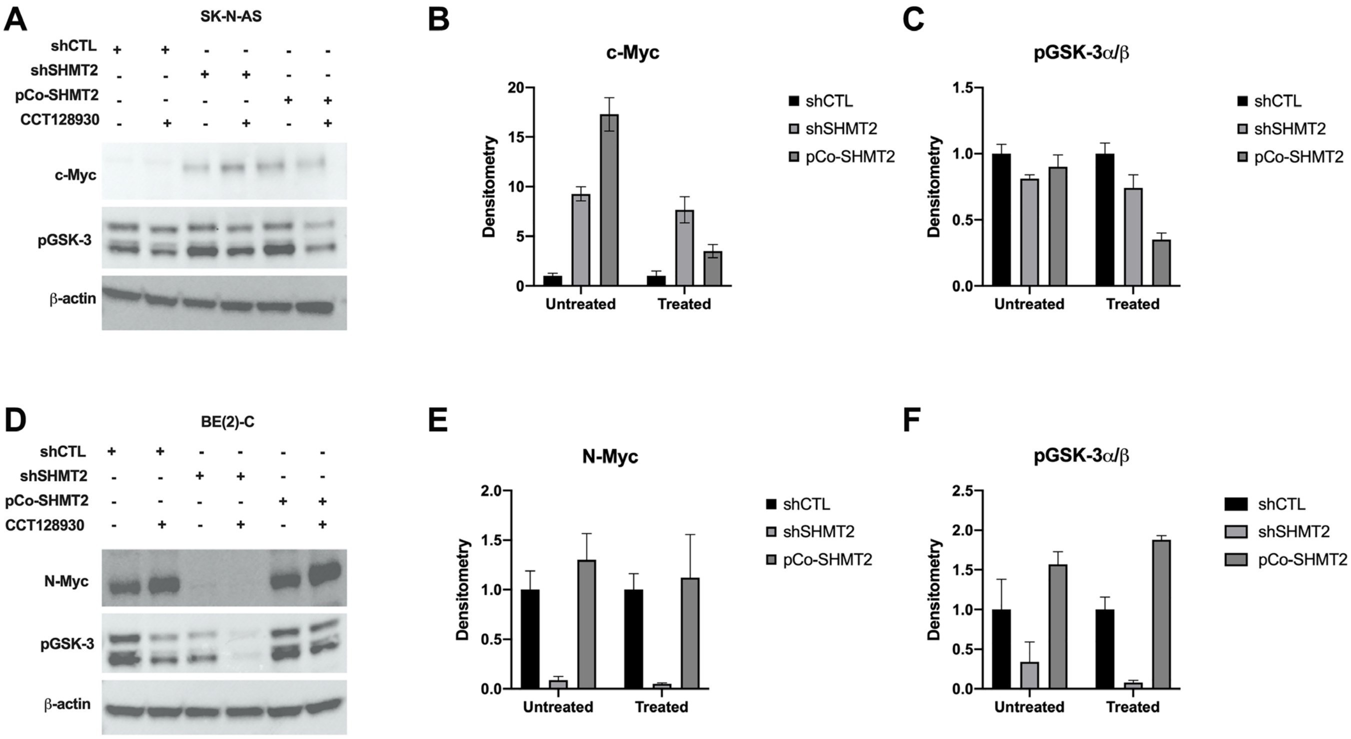


**Supplementary Figure 3: Treatment with an Akt-2 inhibitor confirms SHMT2 silencing and overexpression affect N-Myc/c-Myc via pAkt-2.** The effects of CCT129830 on SK-N-AS and BE(2)-C cells with SHMT2 silencing (shSHMT2) and overexpressing (pCo-SHMT2), compared to control (shCTL). (**A**) Immunoblotting of SK-N-AS cells treated with CCT129830 demonstrating decreased pGSK-3α/β, a down-stream target of Akt-2, confirming successful inhibition of Akt-2 and a decreased effect of SHMT2 silencing and overexpression on c-Myc protein expression. (**B**) Densitometry analysis, reported as a ratio of each protein band density relative to the density of each β-actin control band (protein density: β-actin density), demonstrating a 7.7-fold increase in c-Myc with CCT129830 treatment in SK-N-AS shSHMT2 cells, compared to a 9.3-fold increase without treatment. The effects of SHMT2 overexpression on c-Myc treatment were also decreased with Akt-2 inhibition, demonstrating only a 3.5-fold increase in c-Myc in treated cells, compared to a 17.3-fold increase seen in untreated pCo-SHMT2 cells. (**C**) CCT129830 successfully inhibited Akt-2 in transfected SK-N-AS cells with a 1.4-fold and 2.9-fold decrease in pGSK-3α/β expression in shSHMT2 and pCo-SHMT2 cells, respectively. (**D**) Immunoblotting demonstrating a decrease in the effects of SHMT2 silencing and overexpression on BE(2)-C cells with inhibition of Akt-2. (**E**) Densitometry analysis, reported as a ratio of each protein band density relative to the density of each β-actin control band (protein density: β-actin density), demonstrating an 11-fold decrease in N-Myc with SHMT2 silencing in untreated BE(2)-C cells, compared to a 20-fold decrease in N-Myc with Akt-2 inhibition. SHMT2 overexpression increased N-Myc to a lesser extent with Akt-2 inhibition, with a 1.1-fold increase in treated cells compared to a 1.3-fold increase in N-Myc protein expression with SHMT2 overexpression in untreated cells. (**F**) SHMT2 overexpression increased pGSK-3α/β expression in untreated and treated cells. However, with SHMT2 overexpression in the MYCN-amplified cell line, BE(2)-C, the effects of Akt-2 inhibition were overcome with a 1.9-fold increase in pGSK-3α/β, despite treatment with CCT129830.

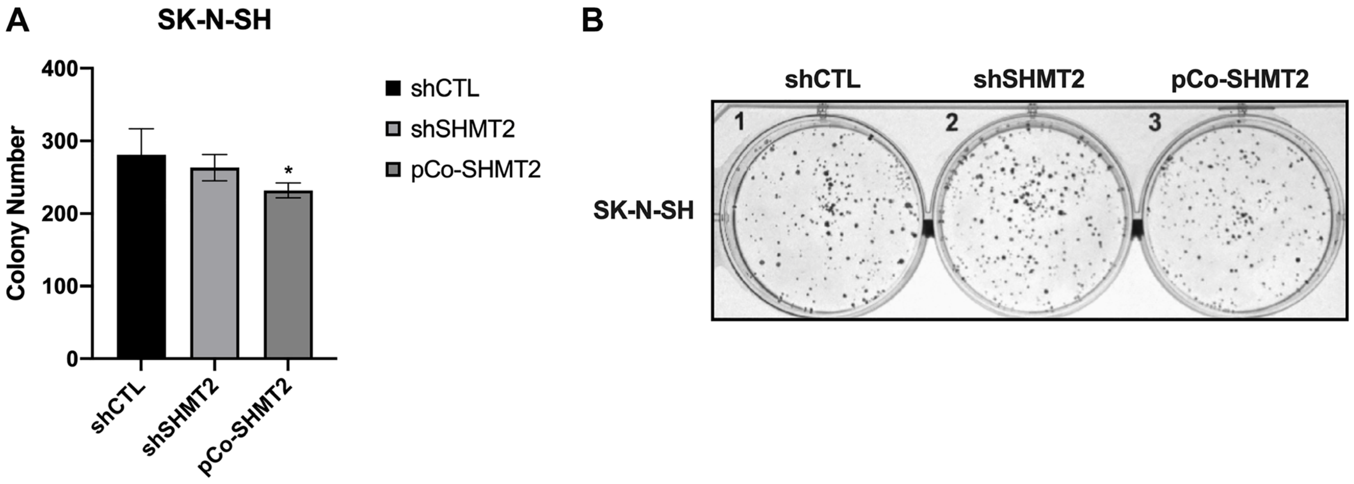


**Supplementary Figure 4: SHMT2 silencing and overexpression did not affect colony formation in the non-*MYCN* -amplified cell line, SK-N-SH.** (**A**) There was no significant difference in colony formation between shCTL and shSHMT2 cells (shCTL 280.7 ± 36 colonies vs shSHMT2 263.1 ± 18 colonies, $p = 0.21$) and there was a significant decrease in colony formation in pCo-SHMT2 cells compared to shCTL (shCTL 280.7 ± 36 colonies vs 231.8 ± 10 colonies, $p = 0.001$). (**B**) Representative image of colony counts. After 7 days, colonies were stained with 5% Crystal Violet and images were taken. As seen, there was a decrease in the number of colonies in shSHMT2, compared to control. SHMT2 overexpression did not increase colony number but increased colony size in the pCo-SHMT2 cells.
